# Fermentation of Chicory Fructo‐Oligosaccharides and Native Inulin by Infant Fecal Microbiota Attenuates Pro‐Inflammatory Responses in Immature Dendritic Cells in an Infant‐Age‐Dependent and Fructan‐Specific Way

**DOI:** 10.1002/mnfr.202000068

**Published:** 2020-06-02

**Authors:** Madelon J. Logtenberg, Renate Akkerman, Ran An, Gerben D. A. Hermes, Bart J. de Haan, Marijke M. Faas, Erwin G. Zoetendal, Henk A. Schols, Paul de Vos

**Affiliations:** ^1^ Laboratory of Food Chemistry Wageningen University and Research Bornse Weilanden 9, 6708 WG Wageningen The Netherlands; ^2^ Immunoendocrinology, Division of Medical Biology, Department of Pathology and Medical Biology University of Groningen and University Medical Centre Groningen Hanzeplein 1, 9700 RB Groningen The Netherlands; ^3^ Laboratory of Microbiology Wageningen University and Research Stippeneng 4, 6708 WE Wageningen The Netherlands

**Keywords:** dendritic cells, in vitro fermentation, infant formula, inulin‐type fructans, microbiota

## Abstract

**Scope:**

Inulin‐type fructans are commonly applied in infant formula to support development of gut microbiota and immunity. These inulin‐type fructans are considered to be fermented by gut microbiota, but it is unknown how fermentation impacts immune modulating capacity and whether the process of fermentation is dependent on the infant's age.

**Methods and results:**

The in vitro fermentation of chicory fructo‐oligosaccharides (FOS) and native inulin are investigated using pooled fecal inocula of two‐ and eight‐week‐old infants. Both inocula primarily utilize the trisaccharides in FOS, while they almost completely utilize native inulin with degree of polymerization (DP) 3–8. Fecal microbiota of eight‐week‐old infants degrades longer chains of native inulin up to DP 16. This correlates with a higher abundance of *Bifidobacterium* and higher production of acetate and lactate after 26 h of fermentation. Fermented FOS and native inulin attenuate pro‐inflammatory cytokines produced by immature dendritic cells (DCs), but profiles and magnitude of attenuation are stronger with native inulin than with FOS.

**Conclusion:**

The findings demonstrate that fermentation of FOS and native inulin is dependent on the infant's age and fructan structure. Fermentation enhances attenuating effects of pro‐inflammatory responses in DCs, which depend mainly on microbial metabolites formed during fermentation.

## Graphical abstract

This in vitro fermentation setup using pooled fecal inocula of two‐ and eight‐week‐old infants showed that chicory fructo‐oligosaccharides and native inulin attenuate pro‐inflammatory responses in infants. The extent of attenuation depends on the microbial metabolites formed during fermentation of fructans, which is affected by the infant's age and corresponding microbial functionality, as well as the size and structure of fructans.

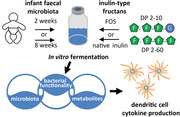

## 1 Introduction

The quality of postnatal nutrition is crucial for health later in life as it is closely intertwined with the microbial colonization of the intestine and the development of the gastrointestinal (GI) tract and immune system.^[^ ^1^, ^2^ ^]^ Exclusive breastfeeding is the gold standard for infants up to 6 months as recommended by the World Health Organization.^[^ ^3^ ^]^ Human milk oligosaccharides (HMOs) are an important component of mother milk, which function as prebiotics and boost immune development for example by supporting the development of the gut immune barrier.^[^ ^4^, ^5^ ^]^ In Europe only approximately 25% of the infants receive exclusive breastfeeding up to an age of six months.^[^ ^6^ ^]^ There is infant formula available for infants for which breast milk is not an option. These formula are supplemented with non‐digestible carbohydrates (NDCs) such as galacto‐oligosaccharides and inulin to mimic the health effects offered by HMOs.^[^ ^7^, ^8^ ^]^

A class of NDCs, commonly used in infant formulas are inulin‐type fructans. Inulin‐type fructans are generally isolated from chicory roots and composed of linear chains of β(2‐1)‐linked fructose monomers with varying degree of polymerization (DP).^[^ ^9^ ^]^ The chains can have either a glucose (GF series) or fructose (F series) moiety at the reducing end, which contributes to structural diversity of the inulin‐type fructans.^[^ ^9^ ^]^ Native inulin predominantly contains the inulin‐type GF series DP2‐60, while the partial hydrolysis product of inulin contains a DP2‐10 GF and F series and is referred to as fructo‐oligosaccharides (FOS—synonym oligofructose).

In recent years evidence has accumulated for the essence of chain length in immune modulation by inulin‐type fructans.^[^ ^10^, ^11^ ^]^ Previous in vitro studies demonstrated direct interaction of inulin‐type fructans with immune cells via pattern recognition receptors (PRRs).^[^ ^11^ ^]^ Inulin‐type fructans with shorter chains resulted in a more anti‐inflammatory cytokine production than inulin‐type fructans with longer chains due to differences in stimulation of PRRs.^[^ ^11^ ^]^ Besides the direct interaction, inulin‐type fructans can also affect immune responses via their fermentation by specific bacteria resulting in the production of, for example, SCFAs.^[^ ^12^, ^13^ ^]^ However, it is unknown how the fermentation of inulin‐type fructans by infant microbiota impacts their immune modulating capacity and whether specific structures of the inulin‐type fructans influence the fermentability and immune effects.

As fermentation capacity is dependent on the age of infants,^[^ ^14^ ^]^ also the impact on immunity might be dependent on the age of the infants. The microbial colonization of the infant gut starts upon birth, when exposure occurs to maternal vaginal and environmental bacteria.^[^ ^15^ ^]^ Initially, due to the high concentration of oxygen present in the gut of the new‐born, aerobic and facultative anaerobic bacteria will colonize.^[^ ^15^ ^]^ These bacteria will consume oxygen and thereby create a more anaerobic environment in the infant gut allowing colonization of strict anaerobic bacteria such as clostridia and bifidobacteria.^[^ ^15^ ^]^ This maturation process most likely influences the utilization of NDCs in the first months of life as different sets of enzymes will become available for the degradation of NDCs depending on the bacteria present in the infant gut.^[^ ^14^ ^]^ In previous studies a clear difference in utilization of HMOs was shown between two and eight week‐old infants.^[^ ^14^ ^]^ Hence, both age groups were included in this study to be able to study the effects of NDCs in this crucial timeframe of infant microbiota development.

In the present study we investigated the fermentation of chicory FOS and native inulin in an in vitro fermentation set‐up using pooled fecal inoculum of two and eight week‐old infants, whose bacterial functionality was found to be largely representative of the fecal inocula of the infant population in general (unpublished data). The ability of the infant fecal microbiota to degrade FOS and native inulin was studied as well as the production of SCFAs and the microbiota composition in time. Fermentation digesta at different time points were incubated with immature dendritic cells derived from the umbilical vein to determine the effect of the fermentation products on cytokine production by immature dendritic cells.

## 2 Experimental Section

### 2.1 Materials

Frutalose OFP (FOS, DP2‐10) and Frutafit IQ (native inulin, DP2‐60) were provided by Sensus (Roosendaal, The Netherlands).

### 2.2 Fermentation of FOS and Native Inulin by Infant Fecal Inoculum

#### 2.2.1 Culture Medium

Standard ileal efflux medium (SIEM; Tritium Microbiology, Veldhoven, The Netherlands) was prepared as described elsewhere with minor modifications.^[^ ^16^ ^]^ Low amount of carbohydrates was added to mimic the infant ideal environment while minimizing background fermentation. The carbohydrate medium component contained (g L^−1^): pectin, 12; xylan, 12; arabinogalactan, 12; amylopectin, 12; and starch, 12 with a final concentration of only 0.24 g L^−1^. The pH was adjusted to 5.8 using 2‐(*N*‐morpholino)ethanesulfonic acid buffer.

#### 2.2.2 Infant Fecal Inoculum

Fecal samples were collected from four exclusively breast‐fed and vaginally born infants. The infants did not receive antibiotic treatment and did not have health issues. At an age of two and eight weeks fecal material was collected from the diaper directly after defecation, transferred to tubes and stored at −20 °C. After the second collection time point, all samples were stored at −80 °C.

The inoculum was prepared as reported elsewhere with some minor modifications.^[^ ^17^ ^]^ After thawing, fecal material of four infants were combined (4 × 0.1 gram) and diluted in 24 mL sterilized NaCl solution (0.9% (*w/v*)) in an anaerobic chamber (gas phase: 81% N_2_, 15% CO_2_, and 4% H_2_) (Bactron 300, Sheldon Manufacturing, Cornelius, USA). Homogenization was performed by the addition of sterile glass beads prior to thorough mixing (2000 rpm). The fecal solution was combined with SIEM medium in a ratio of 5:82 (*v/v*) and used as pooled fecal inoculum, whose bacterial functionality was found to be largely equivalent to that of the individual fecal inocula (unpublished data).

#### 2.2.3 In Vitro Fermentation

Fermentations were performed in duplicate in an anaerobic chamber. Fecal inoculum was combined with SIEM medium containing FOS or native inulin in sterile fermentation flasks in a ratio of 1:10 (*v/v*) with a total volume of 54 mL. The final concentration of FOS and native inulin in the fermentation liquid was 10 mg mL^−1^. The final concentration of SIEM medium carbohydrates only reached 0.24 mg mL^−1^. Fermentation flasks were closed with a rubber stopper that was secured with a metal lid to ensure anoxic conditions. Afterwards flasks were put in an incubator shaker (Innova 40) (37 °C, 100 rpm). At the start and after 14, 20, and 26 h, digesta were collected in triplicate with a syringe. One sample was immediately frozen in liquid nitrogen and stored at −80 °C to preserve the bacteria for later microbial analysis. Both other samples were heated for 5 min in a boiling water bath to inactivate enzymes present. Subsequently they were stored at −20 °C until further analysis.

The following control fermentations were included: 1) inoculum without added FOS and native inulin to monitor background fermentation, 2) FOS and native inulin without inoculum to monitor contamination of the substrate. From these latter controls, only native inulin with SIEM showed some unexpected fermentation after 26 h including inulin utilization and production of substantial amounts of organic acids. Since a later control fermentation using the same set‐up and substrate did not show any sign of fermentation/contamination, it was concluded that the contamination of only the inulin control did not originate from the substrate neither from SIEM. As such, it did not pose any limitations for this study.

### 2.3 Fate of FOS and Native Inulin Upon Fermentation

Degradation of FOS and native inulin during fermentation was analyzed by High Performance Anion Exchange Chromatography (HPAEC). Fermentation samples were diluted until a concentration of 50 µg mL^−1^ and centrifuged (5 min, 15 000 g). Ten µL of sample was injected to an ISC5000 HPLC system (Dionex, Sunnyvale, CA, USA) with a CarboPac PA‐1 column (250 mm × 2 mm ID), a CarboPac PA guard column (25 mm × 2 mm ID) and a ISC5000 ED detector (Dionex) in the pulsed amperometic detector (PAD) mode. Mobile phase A (0.1 m sodium hydroxide) and B (1 m sodium acetate in 0.1 m sodium hydroxide) were used with the following elution profile: 0–25 min, 0–40% B; 25–30 min, 40–100% B; 30–35 min, washing step with 100% B; 35–35.1 min, 100‐0% B; 35.1–50 min, equilibration with 100% A. Flow rate was set at 0.3 mL min^−1^. The data were analyzed using Chromeleon 7.0 (Thermo Scientific).

### 2.4 Production of SCFAs and Other Organic Acids upon Fermentation

In order to quantify the production of SCFAs and organic acids, fermentation samples were subjected to GC and HPLC analysis as described elsewhere^[^ ^18^ ^]^ with minor modifications.

For GC, fermentation samples (1 mg ml^−1^) were mixed in a 2:1 ratio with a solution containing HCl (0.3 m), oxalic acid (0.09 m) and internal standard 2‐ethyl butyric acid (0.45 mg mL^−1^). The mixture was allowed to stand at room temperature for 30 min.

The temperature profile during GC analysis was as follows: 100 °C, maintained for 0.5 min; raised to 180 °C at 8 °C min^−1^, maintained for 1 min; raised to 200 °C at 20 °C min^−1^, maintained for 5 min. Glass wool was inserted in the glass liner of the split injection port to protect the column from contamination.^[^ ^19^ ^]^

### 2.5 Microbial Composition Analysis

#### 2.5.1 DNA Extraction

DNA was extracted from fermentation samples using the repeated beat beating method^[^ ^20^ ^]^ and afterwards purified using the Maxwell 16 Tissue LEV Total RNA Purification Kit Cartridge (AS1220).

#### 2.5.2 PCR Amplification

Microbiota profiling was performed as described previously with some modifications.^[^ ^21^ ^]^ The V5V6 region of 16S ribosomal RNA (rRNA) genes was amplified in triplicate PCR reactions with a unique barcoded primer pair BSF784 (RGGATTAGATACCC) and R1064 (CGACRRCCATGCANACCT). For the t0 samples which contained 1.4‐4.6 ng µL^−1^ DNA, 10 µL of DNA template was used in each reaction. For samples with higher DNA concentrations, 0.7 µL of DNA template was used. Two synthetic communities with known composition were included to evaluate the sequencing performance for reflection of the theoretical composition.^[^ ^22^ ^]^ The communities consisted of 55 16S rRNA gene amplicons of phylotypes associated with the human gut with staggered concentrations typical for the human gut (1) and with relative abundances between 0.001 and 2.49% (2).

#### 2.5.3 Library Preparation and Sequencing

PCR products were purified using HighPrep PCR kit (MagBio Genomics, Alphen aan den Rijn, The Netherlands). Purified amplicons were quantified using Qubit dsDNA BR assay kit (Life Technologies, Leusden, The Netherlands). Seventy unique barcode tags were used in each library.^[^ ^22^ ^]^ Two amplicon pools were formed by combining 200 ng of each barcoded sample and afterwards concentrated to 50 µL volume using the HighPrep PCR kit. Libraries were sent for adapter ligation and sequencing on an Illumina Hiseq2500 instrument (GATC‐Biotech, Konstanz, Germany).

#### 2.5.4 Data Analysis

Processing and analysis of the 16S rRNA gene amplicon sequencing data was carried out using the NG‐Tax pipeline, with default settings^[^ ^22^ ^]^ and R version 3.5.0. Amplicon sequencing variants (ASVs) with a relative abundance below 0.1% were removed. The threshold for taxonomic assignment was set at 80%.

Bifidobacteria were analyzed at the level of individual sequences (Amplicon Sequencing Variants [ASVs]). Species information was obtained with NG‐Tax 2.0^[^ ^23^ ^]^ and SILVA database release 132.^[^ ^24^ ^]^ Tax4Fun2 was used to predict the functional capability of the microbial communities based on 16S rRNA genes.^[^ ^25^ ^]^ Output was visualized in a heatmap with relative abundance of KEGG functional orthologs in the fermentation samples scaled per row and hierarchical clustering using the Ward.D2 algorithm and Euclidean distances using the *pheatmap* R package.^[^ ^26^ ^]^

UniProt Knowledgebase was used to extract all bacterial genes encoding for β‐fructofuranosidase together with the corresponding taxonomic classification. Afterwards Bologna Unified Subcellular Component Annotator (BUSCA) was used to predict the subcellular localization of the enzyme.^[^ ^27^ ^]^

#### 2.5.5 Quantification of Total Bacterial 16 rRNA Gene Copy Number

The total bacterial abundance was determined by quantitative PCR (qPCR) analysis of total 16S rRNA genes using the forward primer 5’‐GTGSTGCAYGGYYGTCGTCA‐3’ and reverse primer 3’‐ACGTCRTCCMCNCCTTCCTC‐5’.^[^ ^28^ ^]^ qPCR amplifications were performed in triplicate and *E. coli* was used as standard for quantification. The qPCR reaction mixture contained 6.25 µL iQ SYBR Green Supermix (Bio‐Rad, Hercules, CA, USA), 200 nm forward and reverse primers, 3.25 uL of Nuclease free water and 2.5 uL of sample DNA (1 ng µL). qPCR cycling conditions and apparatus were used as described elsewhere with a minor modification as an annealing temperature of 52 °C was used instead of 56 °C.^[^ ^29^ ^]^

### 2.6 Stimulation of Dendritic Cells with FOS and Native Inulin Fermentation Samples

#### 2.6.1 Cell Culture and Stimulation

Dendritic cells (DCs) generated from umbilical cord blood CD34^+^ progenitor cells (hematopoietic stem cells) were purchased from MatTek Corporation (Ashland, MA, USA). DCs were freshly thawed and seeded into 96‐well plates at a density of 70 × 10^4^ cells per well and cultured under normal conditions (37 °C, 21% O_2_ and 5% CO_2_) for 24 h according to manufacturer's instructions. After 24 h of culturing, cells were attached, and medium could be replaced. To stimulate DCs with fermentation products of the in vitro fermentation of FOS and native inulin using infant fecal inoculum, bacteria were removed from the fermentation samples by centrifugation (10 min, RT, 12 000 g). Subsequently supernatants were filtered through a 0.2 µm filter and diluted in DC‐MM culture medium (MatTek Corporation, Ashland, MA, USA) containing Polymyxin‐B (50 ng mL^−1^) (Invivogen) at a ratio of 1:10. The pH was set at 7.4 by the addition of 2 n NaOH. DCs were then incubated with 200 µL well^−1^ medium containing 20 µL of fermentation samples for 48 h. After incubation supernatants were collected and stored at −20 °C until further analysis. All experiments were repeated six times.

#### 2.6.2 Assessment of Cytokine Expression

A magnetic Luminex Assay (R&D systems, Bio‐Techne, Minneapolis, USA) was used to measure the levels of monocyte chemoattractant protein‐1 (MCP‐1)/CC chemokine ligand (CCL2), macrophage inflammatory protein 1‐alpha (MIP‐1α)/CCL3, IL‐1β, IL‐6, IL‐10, and tumour necrosis factor alpha (TNFα) in the DC supernatant. The assay was performed according to manufacturer's protocol. Briefly, cytokine standards were resuspended and serial dilutions were prepared. Antibody magnetic bead mix was added to a 96‐well plate. Standards and samples were added and incubated overnight at 4 °C while shaking. After washing the plate three times, detection antibodies were added and the plate was incubated for 30 min at RT while shaking. After incubation, the plate was washed again and incubated with streptavidin‐PE for 30 min at RT while shaking. Finally, the plate was washed again and 100 µL of wash buffer was added to each well. Subsequently, the plate was analyzed using a Luminex 200 System. The data obtained was analysed using the Luminex xPONENT software.

#### 2.6.3 Statistical Analysis

All statistical tests were performed using Prism 8 software (GraphPad, San Diego, CA, USA). Outliers were removed after testing using a Grubbs outlier test (alpha = 0.05). Data are shown as averages and error bars represent standard error of the means. Data was distributed normally and analyzed using a mixed‐effects model (REML) to test for differences between the two and eight weeks groups, followed by a Tukey's multiple comparisons test, to test if the fermentation samples of either 14 or 26 h could induce significantly higher cytokine responses than their corresponding 0h sample. The induced cytokine responses were also compared by the FOS and native inulin samples at the different time points to their corresponding blank samples.

## 3 Results

### 3.1 DP and Structure‐Specific Utilization of FOS and Native Inulin by Fecal Microbiota of Two and Eight Week‐Old Infants

As it has been shown that fecal microbiota of two and eight week‐old infants have a different composition and consequently a different expression of carbohydrate degrading enzymes,^[^ ^14^ ^]^ their ability to ferment FOS (DP2‐10) and native inulin (DP2‐60) were compared. During in vitro fermentation, samples were collected and analyzed with HPAEC to follow the fate of the NDCs. A detailed overview of remaining DP after fermentation of FOS and native inulin is shown in Figure S1, Supporting Information.

**Figure 1:**
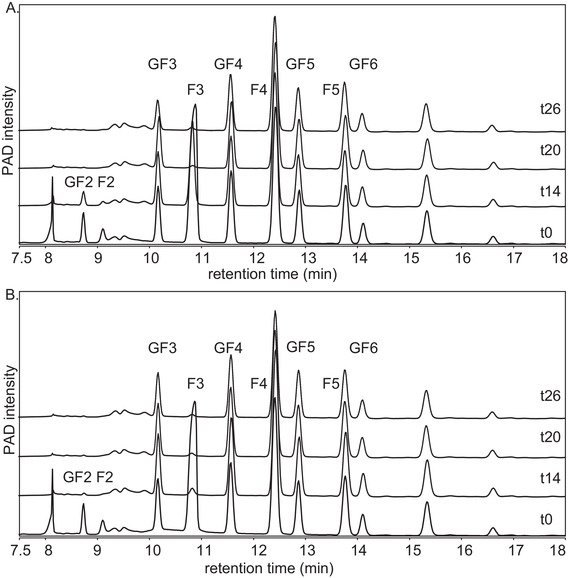
HPAEC profiles of FOS at the start and after 14, 20, and 26 h of fermentation using pooled fecal inoculum of A) two‐ and B) eight‐ week‐old infants. DP and F/GF series are annotated.

Fecal microbiota of both two and eight week‐old infants were able to ferment the trisaccharides with either a terminal fructose or glucose moiety (F and GF series) present in FOS, although eight week‐old infants did this at a higher rate (**Figure** **1**). The fermentation was limited to these DP3 oligomers during the first 14 h of fermentation. Between 14 and 26 h of fermentation also only minor degradation of FOS DP > 3 took place, but fermentation of DP3 oligomers was still most pronounced. After 26 h of fermentation, less than 3% of the DP3 oligomers was remaining, while ≈77% of FOS DP > 3 was still intact with fecal microbiota of two and eight week‐old infants.

**Figure 2:**
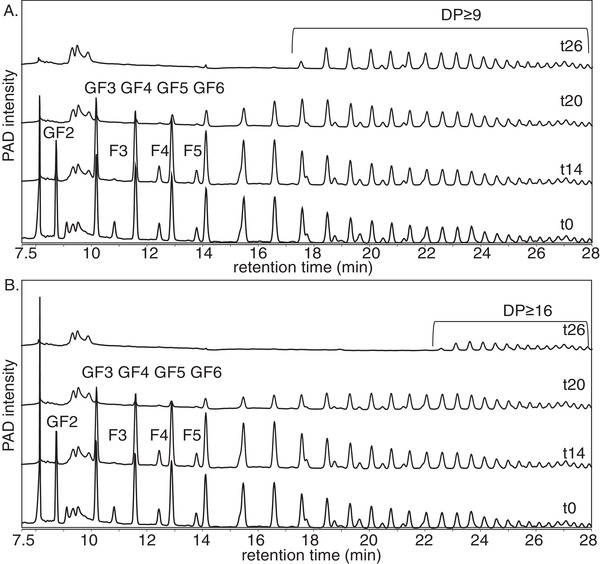
HPAEC profiles of native inulin at the start and after 14, 20, and 26 h of fermentation using pooled fecal inoculum of A) two‐ and B) eight‐week‐old infants. DP and F/GF series are annotated.

Fecal microbiota of two week‐old infants were able to degrade oligomers of native inulin up to DP9 while higher DPs remained virtually intact (**Figure** **2**). There was a relative high recovery of 62% and 99% for respectively DP 9–15 and DP ≥ 16 after 26 h of fermentation. Longer incubation times did not result in significant fermentation of higher DPs (Figure S2, Supporting Information). The fecal microbiota of two week‐old infants had a similar preference for the trisaccharides F3 and GF2 during the first 14 h of fermentation as was observed in the fermentation study with FOS, although the F‐series was less abundant in native inulin compared to FOS.

Fermentation of native inulin using fecal microbiota of two week‐old infants resulted in different degradation patterns compared to the two week‐old infants, as they were able to degrade native inulin of higher DP values. The fecal microbiota of eight week‐old infants was able to completely degrade native inulin up to DP16 and higher DPs were degraded for 40% after 26 h of fermentation. Also, in this case a clear preference for the trisaccharides F3 and GF2 was observed during the first 14 h of fermentation.

### 3.2 Impact of Native Inulin on fecal Microbiota Composition is Different between Two and Eight Week‐Old Infants

**Figure 3:**
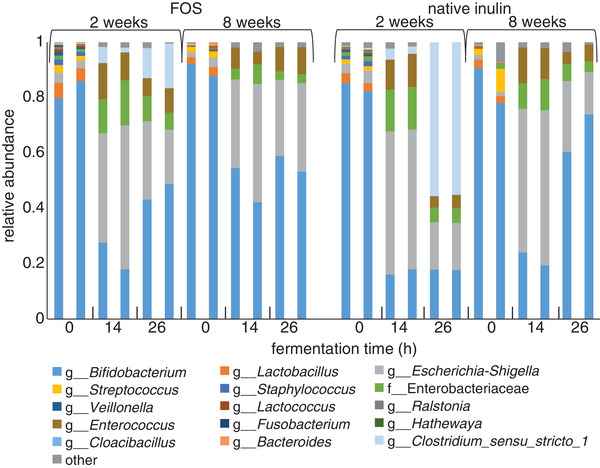
Relative abundance of bacteria at the highest classified taxonomy in duplicate fermentation digesta collected at the start and after 14 and 26 h from in vitro fermentation of FOS and native inulin using pooled fecal inoculum of two and eight week‐old infants with the 15 most abundant bacteria visualized individually and less abundant bacteria summarized as “other”.

To study the impact of FOS and native inulin on the fecal microbiota of two and eight week‐old infants, 16S rRNA gene amplicon sequencing and quantitative PCR (qPCR) of total bacteria was performed. With FOS fermentation, a decrease in relative abundance of *Bifidobacterium* was observed in the first 14 h with both fecal inocula of two and eight week‐old infants (**Figure** **3**, Table S1, Supporting Information) as well as in control fermentations without NDCs (Figure S3, Supporting Information). However, taking into account quantitative PCR analysis of total bacteria demonstrating a 2000‐fold increase in 16S rRNA gene copy numbers between 0 and 14 h of fermentation (Table S2, Supporting Information), fermentation of FOS increased the absolute number of *Bifidobacterium* from 2.8 × 10^6^ to 1.1 × 10^9^ copies in the first 14 h for inocula of 2‐week‐old infants. After 26 h of fermentation 43–49% of the bacteria belonged to the genus *Bifidobacterium*. A control fermentation without NDCs did not result in an increase in *Bifidobacterium* (Figure S3, Supporting Information).

Fermentation of FOS using fecal inoculum of two week‐old infants also resulted in an increase in *Clostridium sensu stricto* resulting in a 11–16% contribution to all bacteria after 26 h. In addition, an enrichment in bacteria belonging to the genera *Enterococcus*, *Escherichia‐Shigella*, and the family Enterobacteriaceae was observed. This enrichment was also observed for control fermentations without added NDCs.

FOS fermented by inoculum of eight week‐old infants also induced an increase in relative abundance of *Bifidobacterium* to 53–59% after 26 h of fermentation. Furthermore, a similar enrichment in bacteria belonging to the genera *Enterococcus* and *Escherichia‐Shigella*, and the family Enterobacteriaceae was observed. However, fermentation of FOS by fecal microbiota of eight week‐old infants did not result in an increase in the relative abundance of *Clostridium sensu stricto*.

Native inulin decreased the relative abundance of *Bifidobacterium* for inocula of two and eight week‐old infants in the first 14 h of fermentation. Again, the qPCR illustrated that this relative decrease did not represent a decrease in absolute numbers of *Bifidobacterium* (Table S2, Supporting Information). After 26 h of fermentation the fecal microbiota of two week‐old infants was for 18% composed of bacteria belonging to the genus *Bifidobacterium. Clostridium sensu stricto* was most abundant; 55% after 26 h of fermentation. Similar to the fermentation of FOS, background fermentation of SIEM medium components was observed.

Native inulin induced a higher increase in relative abundance of *Bifidobacterium* for inocula of eight week‐old infants than for two week‐old infants, but no relative increase in *Clostridium sensu stricto* was observed. After 26 h of fermentation 60–74% of the bacteria belonged to the genus *Bifidobacterium*. Fermentation of native inulin thus showed an age‐dependent stimulation of *Bifidobacterium*, which was not observed for FOS.

### 3.3 Differences in Predicted Functional Capabilities of Microbial Communities from Two and Eight ‐Week‐Old Infants during Native Inulin Fermentation

**Figure 4:**
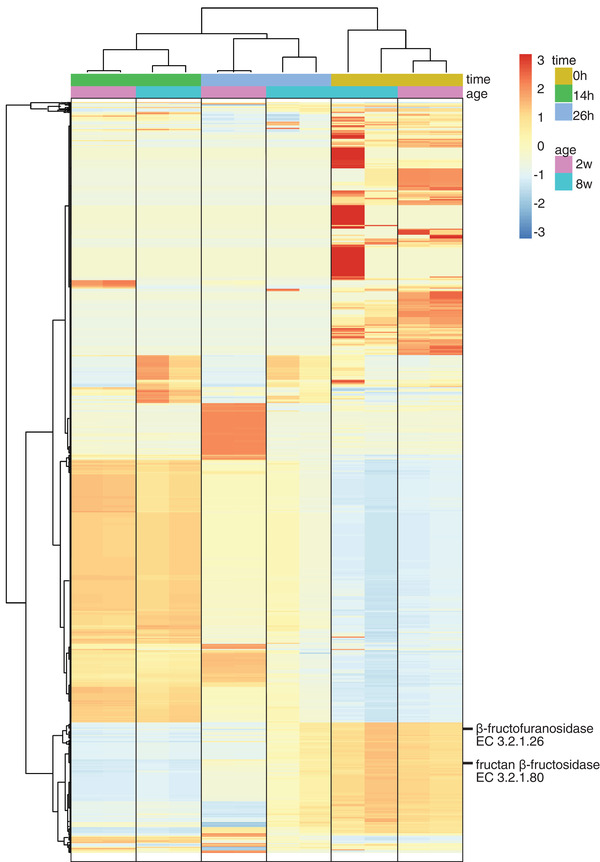
Heatmap of relative abundance of KEGG functional orthologs in fermentation digesta collected at the start and after 14 and 26 h from in vitro fermentation of native inulin using pooled fecal inoculum of two and eight week‐old infants. Colors given by taxa scaling per row with the mean relative abundance in yellow. Hierarchical clustering using Ward.D2 algorithm and Euclidean distances. KEGG functional orthologs necessary for degradation native inulin shown in figure. On top of the heatmap different colors as explained in the legend depict the infant age and fermentation time.

As the degradation kinetics of native inulin differed between the fecal microbiota of two and eight ‐week‐old infants, we questioned whether this might be explained by differences in the predicted functional capability and more specifically in expression of the enzymes β‐fructofuranosidase and fructan β‐fructosidase which are necessary for degradation of native inulin. To this end, an additional analysis was performed to predict the functional capability of the microbial communities based on 16S rRNA genes using Tax4Fun2 (**Figure** **4**). The heatmap shows a clear clustering based on fermentation time and infant age. With native inulin a higher relative abundance of β‐fructofuranosidase and fructan β‐fructosidase was observed for fecal microbiota of eight week‐old infants compared to two week‐old infants.

### 3.4 High Relative Abundance of One Specific ASV in Fermentations of Both FOS and Native Inulin

**Figure 5:**
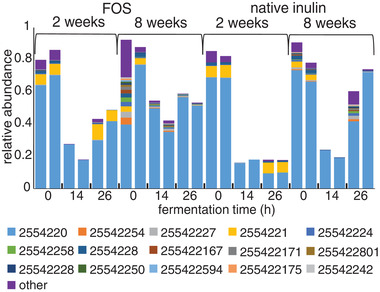
Relative abundance of *Bifidobacterium* Amplicon Sequencing Variants (ASVs) in duplicate fermentation digesta collected at the start and after 14 and 26 h from in vitro fermentation of FOS and native inulin using pooled fecal inoculum of two and eight week‐old infants.

Since mainly *Bifidobacterium* was increased by FOS and native inulin, we performed a higher resolution analysis at the level of their individual sequences (Amplicon Sequencing Variants (ASVs)), which can be considered as a proxy for *Bifidobacterium* species (**Figure** **5**). ASV 2554220 was the most abundant ASV during fermentations of both FOS and native inulin using fecal inoculum of two and eight week‐old infants. Comparison of the sequence with the SILVA database release 132 showed that the sequence was an exact match with species such as *Bifidobacterium breve, Bifidobacterium longum* subspecies *infantis*, and *Bifidobacterium longum* subspecies *longum* (**Table** **1**).

**Table 1:** Taxa matching with ASV 2554220 according to SILVA database release 132

*g_Bifidobacterium* ASV 2554220	
TGGTAGTCCACGCCGTAAACGGTGGATGCTGGATGTGGGGCCCGTTCCACGGGTTCCGTGTCGGAGCTAA	
GTGAACCCGCCCCGAAGGGAAACCCCATCTCTGGGATCGTCGGGAACATGTCAAGCCCAGGTAAGGTTCT	
**Taxa**	**database hits**
*breve*	74
Uncultured organism	45
Human gut metagenome	38
*longum* subspecies *infantis*	34
*longum* subspecies *longum*	11
*longum* subspecies *Lnfantis*_ATCC_15 697_\u003d_JCM_1222_\u003d_DSM_20 088	11
Unidentified	9
Uncultured bacterium	9
*breve*_DSM_20 213_\u003d_JCM_1192	5
*breve*_S27	3
*breve*_ACS‐071‐V‐Sch8b	3
*longum* subspecies *longum*_JDM301	3
*breve*_689b	2
*breve*_UCC2003	2
*breve*_12L	2
*breve*_JCM_7017	2

### 3.5 Extracellular β‐fructofuranosidase not Detected in *Bifidobacterium* Species Present in Fecal Microbiota of Eight Week‐Old Infants

**Figure 6:**
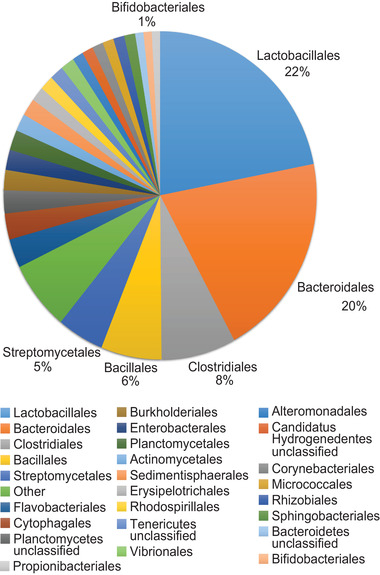
Distribution of extracellular β‐fructofuranosidase genes within sequenced bacterial genomes on order level.

As the expression of extracellular β‐fructofuranosidase could be essential for the utilization of long‐chain inulin, we questioned whether *Bifidobacterium* species present in the fecal microbiota of eight week‐old infants contain the gene that encodes for extracellular β‐fructofuranosidase. The distribution of this gene among bacterial genomes was investigated using UniProtKB and BUSCA (**Figure** **6**). Only three out of 351 bacterial genes encoding for extracellular β‐fructofuranosidase belong to *Bifidobacterium* species, namely to *Bifidobacterium adolescentis, mongoliense*, and *psychraerophilum*. These *Bifidobacterium* species were not detected in native inulin exposed microbiota of two and eight week‐old infants, suggesting that *Bifidobacterium* was not responsible for the utilization of long‐chain inulin.

### 3.6 SCFAs and Organic Acids Are Produced in an Age‐ and Fructan‐Dependent Fashion

**Figure 7:**
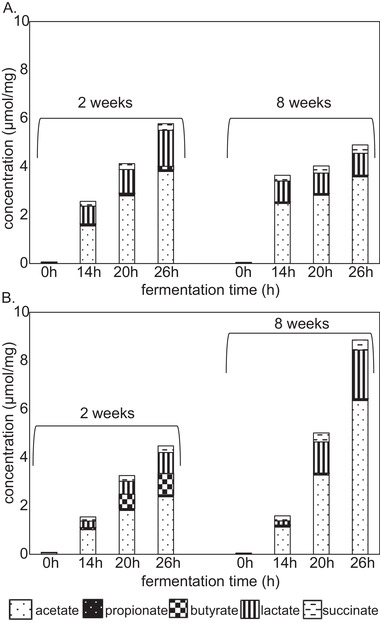
Production of SCFAs, lactacte, and succinate upon fermentation of A) FOS and native B) inulin using pooled fecal inoculum of two‐ and eight‐week‐old infants.

Since SCFAs and other organic acids have shown to be primary fermentation metabolites with important immunomodulatory activity, SCFAs production during fermentation of FOS and native inulin was quantified (**Figure** **7**). Reproducibility was confirmed with duplicate fermentations. A control fermentation without added NDCs showed only minor production of organic acids (Figure S4, Supporting Information).

Fermentation of FOS by fecal inoculum of two week‐old infants resulted in a total organic acid production of 5.8 µmol mg^−1^ after 26 h in a ratio of 66:26:5:2:1 for respectively acetate, lactate, succinate, butyrate, and propionate. Fermentation of FOS by fecal inoculum of eight week‐old infants resulted in a lower amount of total organic acids produced (4.9 µmol mg^−1^). Acetate, lactate, succinate, butyrate and propionate were produced in a ratio of 73:18:7:1:1.

Differences in both total production and composition of organic acids were observed for the fermentation of native inulin using both inocula. The fermentation using fecal inoculum of two week‐old infants resulted in a total organic acid production after 26 h of 4.5 µmol mg^−1^ with a considerable amount of butyrate (0.9 µmol mg^−1^). Acetate, lactate, succinate, butyrate, and propionate were produced in a ratio of 53:19:6:20:2. Fermentation of native inulin by fecal inoculum of eight week‐old infants resulted in a two‐fold higher amount of total organic acids produced, namely 8.9 µmol mg^−1^. Acetate, lactate, succinate, butyrate and propionate were produced in a ratio of 72:22:4:1:1.

### 3.7 Fermentation Digesta of FOS and Native Inulin Induce Infant's Age‐Dependent Cytokine Responses in Dendritic Cells

As in vivo the gastrointestinal tract is aligned with numerous dendritic cells, we studied the impact of digesta of FOS and native inulin fermentation on DC cytokine responses. We compared the impact of digesta of FOS and native inulin fermented by fecal inocula from two and eight week‐old infants. To this end, DCs were incubated with fermentation digesta for 48h and the pro‐inflammatory chemokines and cytokines MCP‐1/CLL2, MIP‐1α/CCL3, IL‐1β, IL‐6, and TNFα as well as the anti‐inflammatory cytokine IL‐10 induced in DCs were measured.

**Figure 8:**
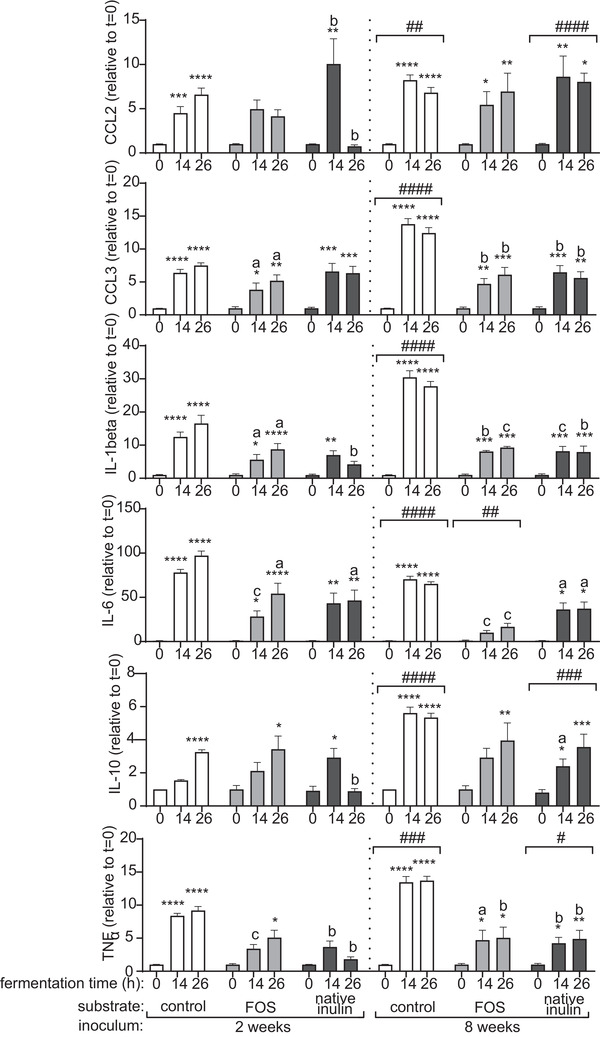
Induced cytokines (fold change of induction by 0 h samples) by digesta of control fermentation, FOS and native inulin fermentation with pooled fecal inocula of two and eight week‐old infants. Stars above bars represent statistical different response compared to the 0h sample (*p < 0.05, **p < 0.01, ***p < 0.001, ****p < 0.0001). Statistical differences between two and eight week groups are indicated above eight weeks graphs (#*p* < 0.05, ##*p* < 0.01, ###*p* < 0.001, ####*p* < 0.0001). Statistical differences between FOS and native inulin samples and their matched time sample of the control fermentation are indicated above bars (a) *p* < 0.05, b) *p* < 0.01, c) *p* < 0.001, d) *p* < 0.0001).

Digesta from control fermentations without added NDCs were incubated with DCs (**Figure** **8**). Both *t* = 14 and *t* = 26 digesta of two and eight week inoculum significantly increased the secretion of most chemokines and cytokines, when compared to *t* = 0. Incubation of DCs with *t* = 14 digesta of the control fermentation with two week inoculum significantly increased the chemokines MCP‐1/CCL2 and MIP‐1α/CCL3, the pro‐inflammatory cytokines IL‐1β, IL‐6, and TNFα but not the anti‐inflammatory cytokine IL‐10. The t = 26 digesta induced a significant increase of all chemokines and cytokines measured. The total upregulation of all chemokines and cytokines was more pronounced with microbiota of eight week‐old infants.

Incubation of DCs with *t* = 14 FOS digesta of two week‐old infants inoculum significantly increased the levels of the chemokine MIP‐1α/CCL3 and cytokines IL‐1β and IL‐6 when compared to digesta of *t* = 0, but levels were significantly lower than the levels induced by *t* = 14 digesta of control fermentations with inocula of two week‐old infants. Incubation of DCs with *t* = 26 FOS digesta fermented with two week‐old infant inoculum increased the same cytokines as well as IL‐10 and TNFα. Again, the levels of MIP‐1α/CCL3, IL‐1β and IL‐6 were lower than levels of these cytokines induced by the *t* = 26 digesta of control fermentations.

DC incubation with digesta of FOS fermented with eight week‐old infant inoculum resulted in different cytokine patterns. DCs incubated with *t* = 14 FOS digesta significantly increased secretion of the chemokines and cytokines MCP‐1/CCL2, MIP‐1α/CCL3, IL‐1β, and TNFα when compared to *t* = 0. However, levels of MIP‐1α/CCL3, IL‐1β and IL‐6 as well as levels of TNFα were significantly lower when compared to cytokine levels induced by *t* = 14 digesta of control fermentations with inocula of eight week‐old infants. With *t* = 26 digesta, levels of MCP‐1/CCL2, MIP‐1α/CCL3, IL‐1β, IL‐10, and TNFα were significantly increased, but levels of MIP‐1α/CCL3, IL‐1β, IL‐6, and TNFα were still significantly lower when compared to cytokine levels induced by *t* = 26 digesta of control fermentations with inocula of eight week‐old infants.

When chemokine and cytokine secretion patterns between DC stimulations with digesta of FOS fermentations with inoculum of two and eight week‐old infants were compared, significant differences between age categories were found for secretion of IL‐6 (*p* < 0.001).

Fermentation of native inulin also demonstrates a clear age‐dependent impact on DC cytokine responses. When DCs where incubated with *t* = 14 native inulin digesta fermented with inoculum of two week‐old infants, levels of the chemokines MCP‐1/CCL2, MIP‐1α/CCL3 IL‐1β, IL‐6 but also of IL‐10 was significantly increased. Notably, the level of MIP‐1α/CCL2 was significantly higher and the level of TNFα was significantly lower than that induced by *t* = 14 digesta of control fermentations. Native inulin *t* = 26 digesta only significantly increased secretion of chemokine MIP‐1α/CCL3 and IL‐6. The levels of the chemokine MIP‐1α/CCL2 and the cytokines IL‐1β, IL‐6, IL‐10, and TNFα were all significantly lower when compared to the *t* = 26 digesta of the control fermentation.

Results were different with eight week inoculum. DCs with native inulin *t* = 14 digesta induced a significant increase in secretion of all chemokines and cytokines measured. However, the levels of MCP‐1/CCL3, IL‐1β, IL‐6, IL‐10, and TNFα were all still significantly lower than the levels of these cytokines induced by the *t* = 14 digesta of the control fermentation. With native inulin *t* = 26 digesta all cytokines significantly increased compared to *t* = 0, but again MCP‐1/CCL3, IL‐1β, IL‐6, and TNFα were still significantly lower than the levels of these cytokines induced by the *t* = 26 digesta of the control fermentation with inoculum of eight week‐old infants.

When two and eight week‐old infants were compared, significant differences were found for secretion of chemokine MIP‐1α/CCL3 (*p* < 0.001), the pro‐inflammatory cytokine TNFα (*p* < 0.05) and the anti‐inflammatory cytokine IL‐10 (*p* < 0.001).

## 4 Discussion

Cow's milk‐based infant formulas are often substituted with NDCs to mimic HMO functionalities. Chicory FOS and native inulin are supplemented to infant formula, but to date minor knowledge is available on structure‐specific utilization of NDCs by infant gut microbiota, production of SCFAs and concomitant immune effects. Therefore, we investigated the degradation of FOS and native inulin by infant fecal microbiota and the effect of the fermentation digesta on dendritic cell responses.

Fermentation of FOS showed selective utilization of the trisaccharides F3 and GF2 in both two and eight week‐old infants, which coincided with an increase in *Bifidobacterium* and an increased production of mainly acetate and lactate. These organic acids are important end products of the bifid shunt, which is a unique hexose metabolism pathway, and consequently suggested to be mainly produced by *Bifidobacterium*.^[^ ^30^ ^]^ The stimulation of *Bifidobacterium* by FOS corroborates findings of a previous in vivo study^[^ ^31^ ^]^ in infants receiving FOS‐supplemented infant formula. The utilization of FOS by *Bifidobacterium* is attributed to several properties such as expression of the gene that encodes for β‐fructofuranosidase, which is responsible for the cleavage of the β (2‐1) bonds between the fructose units in FOS.^[^ ^32^ ^]^

Our data suggest that fermentation of FOS mostly benefit *Bifidobacterium breve*, which is commonly found in the infant gut.^[^ ^33^ ^]^ In contrast to other *Bifidobacterium* species, *Bifidobacterium breve* showed to have a similar preference for FOS DP3.^[^ ^34^ ^]^ DP3 was also first fermented when native inulin was used as substrate, but here, in our study, DP 2–8 F and GF series were degraded more extensively. This can be explained by the presence of higher DPs and the relatively low abundance of DP3 (8% versus 32%) in native inulin, what could have stimulated a broader range of bacterial enzymes.

Native inulin was fermented differently by fecal microbiota of two and eight week‐old infants. The fecal microbiota of eight week‐old infants fermented up to a higher DP than the two week‐old infants. This correlated with a higher relative increase in *Bifidobacterium* and higher production of acetate and lactate. Whereas most *Bifidobacterium* species utilize FOS by intracellular uptake and degradation, this uptake has been suggested to be lower for longer inulin oligosaccharides due to their larger size.^[^ ^35^ ^]^ Previous studies have shown that the growth of *Bifidobacterium* on inulin is dependent on the transcription of extracellular β‐fructofuranosidase.^[^ ^36^ ^]^

Extracellular β‐fructofuranosidase is predominantly present among bacterial orders such as Bacteroidales and Lactobacillales. *Bifidobacterium* species which contain the gene encoding for extracellular β‐fructofuranosidase were not detected in fecal microbiota of both two and eight week‐old infants. Therefore, it is possible that cross‐feeding between *Bifidobacterium* and other bacteria occurred during degradation of higher DPs by the fecal microbiota of eight week‐old infants. Similar cross‐feeding is reported between *Lactobacillus salivarius* and *Lactobacillus paracasei*, where the latter uses an exo‐inulinase to degrade long‐chain inulin.^[^ ^37^ ^]^ However, it cannot be excluded that certain *Bifidobacterium* species can internalize native inulin DP 9–16 by ABC transport systems, which was reported for *Roseburia inulinivorans*.^[^ ^32^ ^]^

Along with *Bifidobacterium*, also a relative increase in *Clostridium sensu stricto I* was observed during fermentation of FOS and native inulin by two week‐old infants, which corresponded with an increased production of butyrate. In contrast to *Bifidobacterium*, several *Clostridium* species ferment hexoses through the Embden–Meyerhof–Parnas or pentose phosphate pathway with butyrate being one of the important end products.^[^ ^38^ ^]^ Although *Clostridium* can utilize FOS and inulin,^[^ ^39^ ^]^ the increase in *Clostridium sensu stricto* in the control fermentation without added NDCs indicate their increase is due to fermentation of SIEM medium components.

Notably, more bacteria were stimulated by SIEM medium. For all fermentations an enrichment in bacteria belonging to the genera *Enterococcus* and *Escherichia–Shigella* and the family Enterobacteriaceae was observed in the first 14 h of fermentation. These bacteria were reported to grow well on peptides and amino acids which are highly abundant in SIEM medium: bactopeptone (24 g L^−1^), casein (24 g L^−1^), ox‐bile (0.4 g L^−1^) and cysteine (0.16 g L^−1^).^[^ ^40^ ^]^ As the enrichment was also observed for control fermentations without added NDCs, it can be concluded that their growth was stimulated by these medium components.

Fermentation as such had a strong impact on cytokine production by immature umbilical vein derived dendritic cells. The control fermentation digests without added NDCs with both two and eight week‐old infant inoculum induced high levels of the chemokines and cytokines MCP‐1/CLL2, MIP‐1α/CCL3, IL‐1β, IL‐6, and TNFα as well as the anti‐inflammatory cytokine IL‐10. *T* = 0 digesta only induced minor cytokine production in DCs, indicating that products formed during fermentation are responsible for the observed effects. As production of SCFAs in the control fermentation is very low, we assume that specific bacterial products such as adenosine triphosphate, lipoteichoic acid, polysaccharide A, peptidoglycan, exopolysaccharide, and RNA/DNA sequences produced by bacteria during fermentation are responsible for these immune stimulating effects.^[^ ^41^ ^]^ These bacterial products interact with specialized immune receptors on immune cells such as Toll like receptors that are expressed on immune cells such as dendritic cells.^[^ ^41^ ^]^

Fermentation of FOS and native inulin reduced the induced enhancement of cytokines by control fermentation. For FOS we found a clear attenuation of the production of multiple pro‐inflammatory cytokines compared to the control fermentation. This phenomenon could possibly be explained by the production of SCFAs upon fermentation as shown here and by others.^[^ ^42^ ^]^ A very pronounced effect was observed for IL‐6 secretion by DCs, which was virtually gone with FOS digesta of fecal inoculum of eight week‐old infants and profoundly reduced with fecal inoculum of two week‐old infants fermenting FOS. For other cytokines no significant differences were observed between FOS and the control fermentation, which correlated with the similarities in changes in microbiota composition and functionality between fecal microbiota of two and eight week‐old infants upon fermentation of FOS.

Native inulin also attenuated the production of pro‐inflammatory cytokines, however the profiles and magnitude of attenuation were different than with FOS. Surprisingly, for the digesta of two week‐old infants this attenuation was more pronounced after 26 h of fermentation for the cytokines MCP‐1/CCL2, IL‐1β, IL‐10, and TNFα. This specific native inulin‐26h fermentation sample was also found to have a high relative increase in *Clostridium sensu stricto I* and increased production of butyrate. Butyrate is known to be an immune attenuating SCFA that acts via GPR109a receptor present on dendritic cells.^[^ ^43^ ^]^ Previous studies also found that butyrate altered the production of pro‐inflammatory cytokines as well as the anti‐inflammatory cytokine IL‐10.^[^ ^44^ ^]^ In addition, it was found that butyrate delayed maturation of DCs and thereby influences T‐cell polarization.^[^ ^45^ ^]^ The generation of butyrate from fermented native inulin might therefore beneficially influence immune development as butyrate can guide immune polarization.^[^ ^45^ ^]^

## 5 Concluding Remarks

This study provides insight into the complex relation between the structure of fructans, the fecal microbiota composition, fermentation capacity, SCFAs production and immune effects in infants. Our data shows that differences in microbial functionality between two and eight week‐old infants impacts fermentation of fructans and therewith the extent to which it performs attenuation of pro‐inflammatory responses in immature DCs. Our findings may contribute to design and tailoring of NDCs mixtures for substitution of infant formulas to meet the needs of infants of different ages and state of inflammation.

## Conflict of Interest

The authors declare no conflict of interest.

## Author Contributions

M.J.L. and R.Ak. contributed equally to this work. M.J.L, R.Ak., H.A.S., and P.D.V. designed the study. M.J.L performed the fermentation experiments. R.Ak. performed the cell‐based experiments. M.J.L performed the microbiota analysis, assisted by R.An, G.D.A.H., and E.Z. M.M.F. and B.J.D.H. assisted with the cell‐based experiments. M.J.L, R.Ak., H.A.S., and P.D.V. wrote the manuscript. All authors have revised and improved the manuscript.

## Supporting information

Supporting Information
