# Supplementary material for: Fermentation of Chicory Fructo‐Oligosaccharides and Native Inulin by Infant Fecal Microbiota Attenuates Pro‐Inflammatory Responses in Immature Dendritic Cells in an Infant‐Age‐Dependent and Fructan‐Specific Way
Source: Mol Nutr Food Res. 2020 Jun 2;64(13):2000068. doi: 10.1002/mnfr.202000068 (PMC7378940; doi:10.1002/mnfr.202000068)
Supplement: Supplementary file 1 — Supporting Information [file MNFR-64-2000068-s001.pdf]

Fermentation of chicory fructo-oligosaccharides and native inulin by infant faecal microbiota attenuates pro-inflammatory responses in immature dendritic cells in an infant-age and fructan-specific way

Madelon J. Logtenberg, Renate Akkerman, Ran An, Gerben D.A. Hermes, Bart J. de Haan, Marijke M. Faas, Erwin G. Zoetendal, Henk A. Schols and Paul de Vos

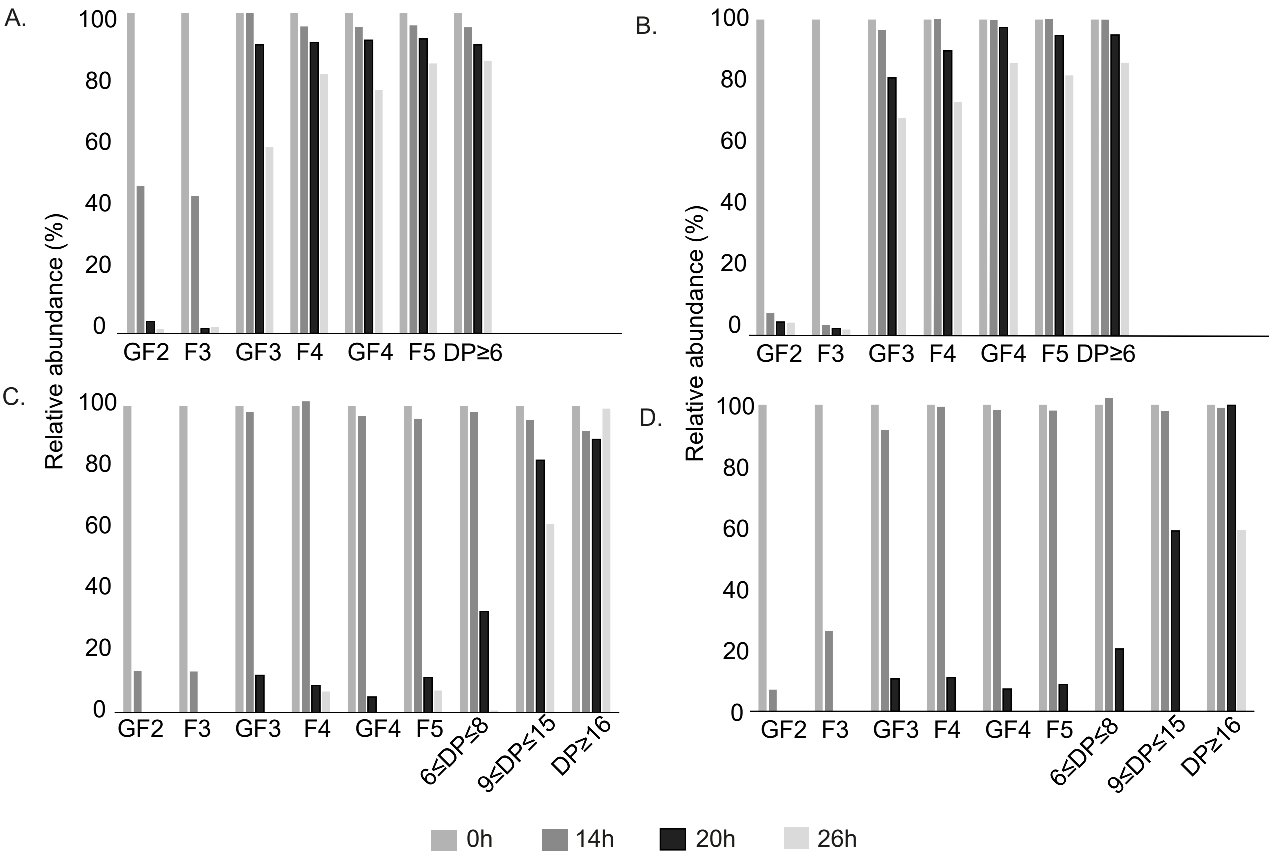

Figure S1. Percentages of remaining compounds with different DP present in FOS (A, B) and native inulin (C, D) during *in vitro* fermentation using pooled faecal inoculum of 2- (A, C) and 8- (B, D) week-old infants.
Analysis was performed by HPAEC-PAD. Concentrations per compound/DP range in the original FOS and native inulin mixture were set to 100%.

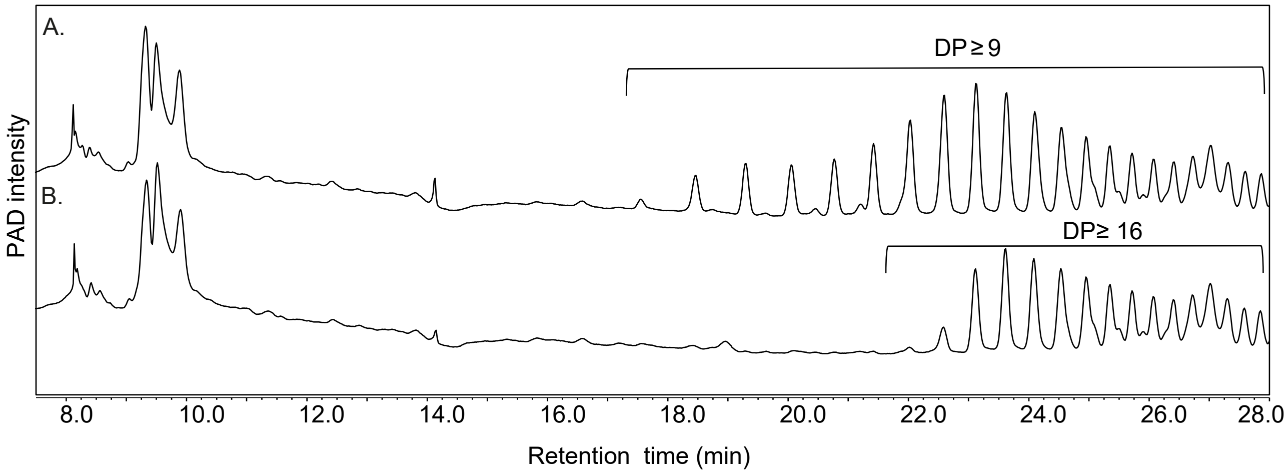

Figure S2. HPAEC profiles of native inulin after 36 hours of *in vitro* fermentation using pooled faecal inoculum of 2- (A) and 8- (B) week-old infants.

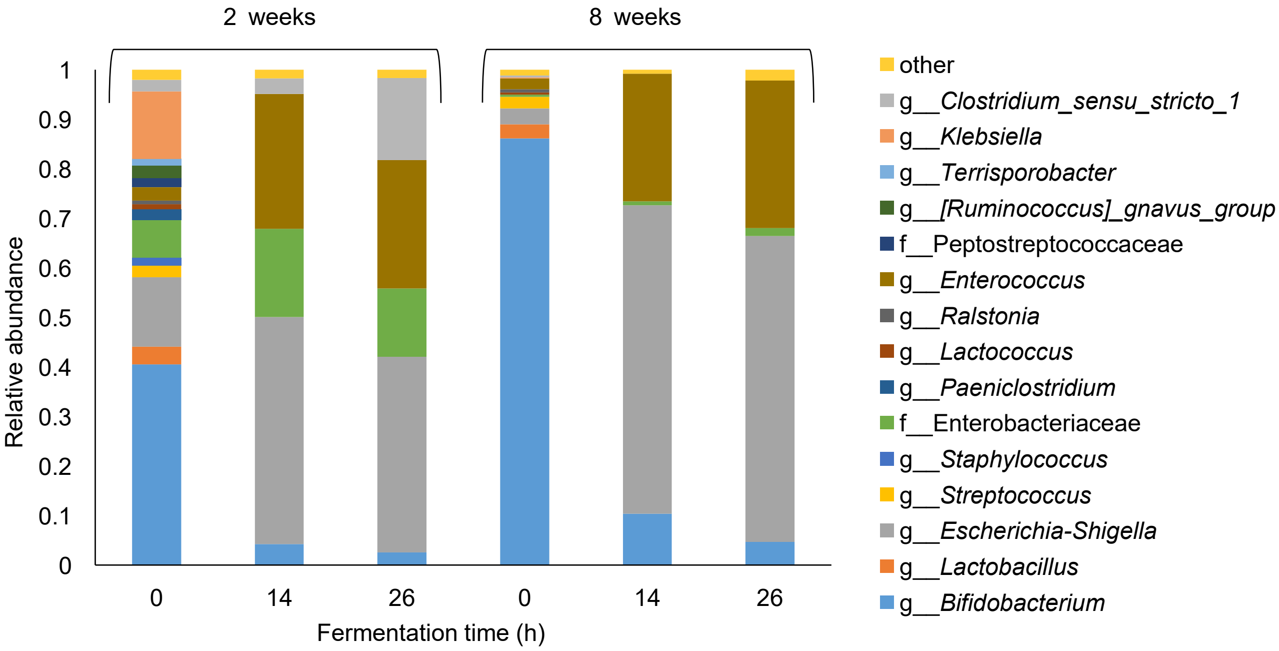

Figure S3. Relative abundance of bacteria at the highest classified taxonomy in fermentation digesta collected at the start and after 14 and 26 hours from *in vitro* fermentation containing solely SIEM medium and faecal inoculum of 2- and 8- week-old infants.

Table S1. Relative abundance of bacteria at the highest classified taxonomy in duplicate fermentation digesta ([a],[b]) collected at the start and after 14 and 26 h from *in vitro* fermentation of FOS and native inulin using pooled faecal inoculum of 2- and 8-week-old infants with the 15 most abundant bacteria individually and less abundant bacteria summarized as 'other' with control: control fermentation without added substrate.

taxonomic classification	FOS												native inulin												control					
	2 weeks						8 weeks						2 weeks						8 weeks						2 weeks			8 weeks		
	0[a]	0[b]	14[a]	14[b]	26[a]	26[b]	0[a]	0[b]	14[a]	14[b]	26[a]	26[b]	0[a]	0[b]	14[a]	14[b]	26[a]	26[b]	0[a]	0[b]	14[a]	14[b]	26[a]	26[b]	0	14	26	0	14	26
Bifidobacterium	79.9	86.0	27.7	18.1	43.2	48.9	92.3	87.8	54.6	42.3	58.9	53.4	85.3	82.3	16.1	18.0	17.9	17.8	90.7	78.1	24.1	19.4	60.4	74.0	40.5	4.2	2.6	86.2	10.4	4.7
Lactobacillus	5.5	4.5	0.0	0.0	0.0	0.0	2.4	3.3	0.0	0.0	0.0	0.0	3.5	3.0	0.0	0.0	0.0	0.0	3.1	2.5	0.0	0.0	0.0	0.0	3.6	0.0	0.0	2.8	0.0	0.0
Escherichia-Shigella	3.6	2.6	39.5	51.9	28.3	19.6	1.6	3.4	32.0	42.7	27.6	31.9	3.4	4.6	51.7	50.5	17.1	17.0	1.7	1.4	51.9	56.1	25.6	15.3	14.0	45.8	39.4	3.2	62.2	61.7
Streptococcus	2.8	1.8	0.0	0.0	0.0	0.0	2.1	2.2	0.0	0.0	0.0	0.0	1.6	1.6	0.0	0.0	0.0	0.0	2.3	8.5	0.0	0.0	0.0	0.0	2.4	0.0	0.0	2.3	0.0	0.0
Staphylococcus	2.1	1.4	0.0	0.0	0.0	0.0	0.0	0.0	0.0	0.0	0.0	0.0	1.3	1.8	0.0	0.0	0.0	0.0	0.0	0.0	0.0	0.0	0.0	0.0	1.5	0.0	0.0	0.0	0.0	0.0
Enterobacteriaceae	1.4	1.0	12.3	16.3	9.1	6.1	0.0	0.3	4.0	7.0	3.0	3.2	1.3	1.8	15.1	15.3	5.3	5.5	0.1	1.8	9.2	11.3	6.2	3.7	7.6	17.9	13.8	0.4	0.8	1.6
Veillonella	1.3	0.6	0.0	0.0	0.0	0.0	0.0	0.0	0.0	0.0	0.0	0.0	0.6	0.7	0.0	0.0	0.0	0.0	0.0	0.0	0.0	0.0	0.0	0.0	0.0	0.0	0.0	0.0	0.0	0.0
Lactococcus	1.0	0.5	0.0	0.0	0.0	0.0	0.0	0.5	0.0	0.0	0.0	0.0	0.4	0.5	0.0	0.0	0.0	0.0	0.3	0.0	0.0	0.0	0.0	0.0	1.0	0.0	0.0	0.4	0.0	0.0
Ralstonia	0.6	0.3	0.0	0.0	0.0	0.0	0.2	0.5	0.0	0.0	0.0	0.0	0.5	0.5	0.0	0.0	0.0	0.0	0.2	0.0	0.0	0.0	0.0	0.0	0.8	0.0	0.0	0.7	0.0	0.0
Enterococcus	0.3	0.3	13.1	10.2	6.5	8.9	0.3	0.5	7.6	4.7	8.8	9.8	0.2	0.4	10.8	12.0	4.1	4.7	0.3	0.2	12.9	11.2	4.4	6.0	2.7	27.2	25.9	2.2	25.8	29.7
Fusobacterium	0.3	0.0	0.0	0.0	0.0	0.0	0.0	0.0	0.0	0.0	0.0	0.0	0.0	0.0	0.0	0.0	0.0	0.0	0.0	0.0	0.0	0.0	0.0	0.0	0.0	0.0	0.0	0.0	0.0	0.0
Hathewaya	0.3	0.1	0.0	0.0	0.0	0.0	0.0	0.2	0.0	0.0	0.0	0.0	0.2	0.2	0.0	0.0	0.0	0.0	0.0	0.0	0.0	0.0	0.0	0.0	0.0	0.0	0.0	0.0	0.0	0.0
Cloacibacillus	0.3	0.0	0.0	0.0	0.0	0.0	0.0	0.0	0.0	0.0	0.0	0.0	0.0	0.0	0.0	0.0	0.0	0.0	0.0	0.0	0.0	0.0	0.0	0.0	0.0	0.0	0.0	0.0	0.0	0.0
Bacteroides	0.3	0.1	0.0	0.0	0.0	0.0	0.3	1.3	0.0	0.0	0.0	0.0	0.1	0.2	0.0	0.0	0.0	0.0	0.4	0.2	0.0	0.0	0.0	0.0	0.0	0.0	0.0	0.0	0.0	0.0
Clostridium sensu stricto 1	0.2	0.2	5.9	1.7	10.8	16.2	0.3	0.0	0.0	0.0	0.0	0.0	0.2	0.6	3.9	2.6	55.6	55.1	0.0	0.0	0.0	0.0	0.0	0.0	2.3	3.1	16.6	0.4	0.0	0.0
other	0.2	0.3	1.6	1.9	2.1	0.4	0.5	0.1	1.9	3.4	1.6	1.8	1.3	2.0	2.3	1.5	0.0	0.0	0.9	7.3	1.9	2.1	3.4	0.8	23.6	1.8	1.7	1.4	0.8	2.2

Table S2. Amount of DNA copies (log) in fermentation digesta collected at the start and after 14 and 26 hours from *in vitro* fermentation of FOS (A) and native inulin (B) using faecal inoculum of 2- and 8-week-old infants with a,b: biological replicate, *: control fermentation without inoculum. Control fermentations without added NDCs are displayed in C.

A.

age	timepoint	DNA copies (log/ml fermentation digesta)
2	0	6.38[a]
2	0	6.64[b]
2	14	9.64[a]
2	14	9.75[b]
2	26	9.74[a]
2	26	9.93[b]
8	0	6.60[a]
8	0	6.66[b]
8	14	9.84[a]
8	14	9.93[b]
8	26	9.74[a]
8	26	9.68[b]
*	0	6.33
*	14	6.03
*	26	8.67

B.

age	timepoint	DNA copies (log/ml fermentation digesta)
2	0	6.55[a]
2	0	6.62[b]
2	14	10.00[a]
2	14	9.89[b]
2	26	10.25[a]
2	26	10.16[b]
8	0	6.56[a]
8	0	7.02[b]
8	14	9.85[a]
8	14	9.84[b]
8	26	10.00[a]
8	26	9.91[b]
*	0	6.63
*	14	9.73
*	26	9.86

C.

age	timepoint	DNA copies (log/ml fermentation digesta)
2	0	6.66
2	14	10.00
2	26	9.90
8	0	6.58
8	14	9.73
8	26	9.96

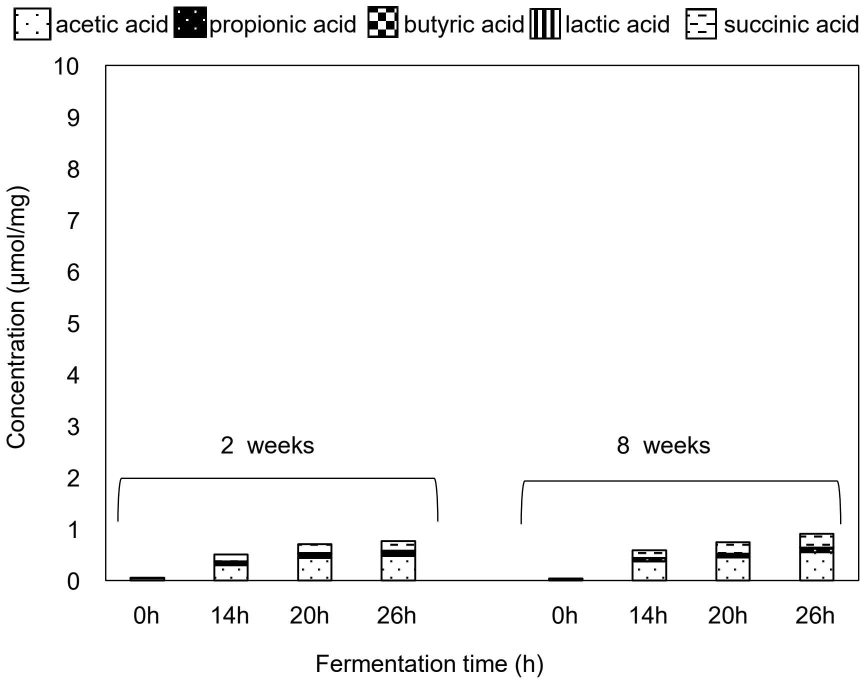

Figure S4. Production of SCFAs, lactic acid and succinic acid upon *in vitro* fermentation containing solely infant faecal inoculum and SIEM medium.
